# Leptin in Dairy Cows: Metabolic Adaptation, Reproductive Function, and Health Applications

**DOI:** 10.3390/life16060987

**Published:** 2026-06-11

**Authors:** Marcelo Martinez-Barbitta, Andrea Biagini, Egidia Costanzi, Gabriella Guelfi, Margherita Maranesi, Juan García-Díez, Cristina Saraiva, Musafiri Karama, Saeed El-Ashram, Ebtesam Al-Olayan, Beniamino Cenci-Goga, Massimo Zerani

**Affiliations:** 1 Sistema Reproductivo Veterinario Integral Uruguay (SRVI_UY), Nueva Helvecia 70300, Uruguay; 2 Dottorato di Ricerca in Patologie Infiammatorie ed Infettive, Strategie Terapeutiche e Biodiritto, Università di Perugia, 06121 Perugia, Italy; andrea.biagini@dottorandi.unipg.it; 3 Dipartimento di Medicina Veterinaria, Università di Perugia, 06121 Perugia, Italy; egidia.costanzi@unipg.it (E.C.); gabriella.guelfi@unipg.it (G.G.); margherita.maranesi@unipg.it (M.M.); massimo.zerani@unipg.it (M.Z.); 4 Centro de Investigação Veterinária e Animal, Universidade de Trás-os-Montes e Alto Douro, Quinta de Prados, 5000-801 Vila Real, Portugal; juangarciadiez@utad.pt (J.G.-D.); crisarai@utad.pt (C.S.); 5 Departamento de Ciências Veterinárias, Escola de Ciências Agrícolas e Veterinárias, Universidade de Trás-os-Montes e Alto Douro, Quinta de Prados, 5000-801 Vila Real, Portugal; 6 Laboratório de Ciências Animais e Veterinárias, Universidade de Trás-os-Montes e Alto Douro, Quinta de Prados, 5000-801 Vila Real, Portugal; 7 Faculty of Veterinary Science, Department of Paraclinical Sciences, University of Pretoria, Pretoria 0110, South Africa; musafiri.karama@up.ac.za; 8 Zoology Department, Faculty of Science, Kafr El-Sheikh University, Kafr El-Sheikh 33516, Egypt; saeed_elashram@yahoo.com; 9 Department of Zoology, College of Science, King Saud University, Riyadh P.O. Box 2454, Saudi Arabia; eolayan@ksu.edu.sa

**Keywords:** leptin, adipokines, dairy cow, energy balance, transition period, reproductive axis, ovarian function, uterine health, metabolic diseases, biomarkers, puberty, feed efficiency, genomic selection

## Abstract

Leptin (LEP) is an adipocyte-derived cytokine that integrates nutritional status, metabolism, and reproduction in cattle, with particular relevance for modern high-producing dairy cows. In ruminants, LEP and its receptors are widely expressed in metabolic and reproductive tissues, including adipose tissue, liver, hypothalamus, pituitary, ovary, uterus, and placenta, where LEP modulates energy homeostasis, neuroendocrine function, and local tissue responses. Changes in circulating LEP concentrations during the transition period reflect changes in body fat reserve, insulin and GH-IGF-1 dynamics, thyroid hormones, and inflammation and contribute to coordinated metabolic adaptations supporting the onset of lactation. At the reproductive level, LEP influences the hypothalamic–pituitary–gonadal axis, affects the pulsatility of luteinizing hormone (LH) under nutritional stress, and exerts direct effects on ovarian steroidogenesis, folliculogenesis, oocyte competence, embryo development, and uterine immune function. New evidence also links LEP profiles to major peripartum disorders, including subclinical ketosis, insulin resistance, postpartum ovarian inactivity, and uterine inflammatory diseases, and emphasises its potential as part of a panel evaluating the risk of metabolic and reproductive disorders. Furthermore, polymorphisms within the bovine LEP gene and its signalling network have been associated with milk production, feed efficiency, body condition, and fertility traits, suggesting opportunities to incorporate markers into genomic selection schemes aimed at improving robustness and reproductive performance. This review summarises current knowledge on LEP biology in cattle, with an emphasis on dairy cows, and discusses perspectives on translating this information into practical tools for nutritional management, health monitoring, and genetic improvement in bovine production systems.

## 1. Introduction

### 1.1. Historical Background of Leptin

Adipose tissue is considered the largest endocrine tissue in mammals [1], secreting a large number of bioactive compounds, called adipokines. These are divided into adipose-specific cytokines directly released by adipocytes (adiponectin, leptin, omentin, resistin, and visfatin) and non-adipose-specific cytokines secreted by various cell types [2].

The modern concept of adipose tissue as an endocrine organ that regulates energy balance originates from Kennedy’s lipostasis hypothesis [3], which in the 1950s proposed that body fat mass is defended by a feedback system in which a signal proportional to adiposity informs hypothalamic centres controlling appetite and energy expenditure. This idea received crucial experimental support from Coleman’s parabiosis studies [4] in the 1960s–1970s, in which cross-circulation between obese *ob/ob* and lean mice showed that *ob/ob* animals lack a circulating satiety factor, while similar experiments with *db/db* mice demonstrated that they produce this factor in excess but are insensitive to it, implying a humoral signal derived from adipose tissue and a distinct receptor. Decades later, the Friedman research group [5] identified the ob gene in ob/ob mice and showed that it encodes leptin (LEP), an adipocyte-derived hormone whose administration normalises food intake, body weight, and metabolic parameters in LEP-deficient mice, thus providing the molecular identity of the adiposity signal envisioned by Kennedy and functionally revealed by Coleman’s parabiosis experiments and establishing LEP as a key link between fat stores and central regulation of energy homeostasis.

These seminal observations provided the conceptual framework for subsequent studies in domestic species, leading to recognition of LEP as a central integrator of nutritional status, metabolism, and reproduction, particularly relevant in high-yielding dairy cows [6].

Bauman and Currie [7] introduced the concept of homeorhesis to describe coordinated endocrine adaptations that prioritise nutrient partition during demanding physiological states such as pregnancy and lactation [8], a framework for interpreting LEP signalling in ruminants. This model supported subsequent research on growth hormone (GH) as a major regulator in lactating ruminants [9], the dynamics of the GH-insulin-like growth factor-1 (IGF-1) axis at lactation onset, and recently on tissue-derived hormones [10,11,12], among which LEP has acquired a fundamental role in the physiology of ruminants.

### 1.2. Leptin in Ruminant Physiology

Leptin acts in an autocrine, paracrine, and endocrine manner and, in ruminants, contributes primarily to long-term metabolic adaptation rather than to acute suppression of food intake [13]. In dairy cows, LEP is best seen as an adiposity-related signal that integrates information on body fat mass, energy balance, and inflammatory tone and feeds this information back to the central and peripheral tissues involved in the partition of nutrients, thyroid function, and immune activity [14,15,16,17,18]. Unlike rodents and humans, where a decrease in LEP is a powerful orexigenic signal, physiological fluctuations in circulating LEP in cattle have limited impact on short-term food intake, and available data do not support a primary role for LEP as an acute satiety factor under field conditions [19,20,21,22,23,24,25]. A clear circadian rhythm of LEP secretion has not been consistently demonstrated in ruminants, although feeding time and general nutritional management can influence circulating adipokines and other metabolic hormones [26]. In dairy cows, LEP secretion is regulated by chronic mechanisms, reflecting body fat reserves, and by more acute mechanisms related to feed intake and energy balance, and these combined regulatory inputs shape LEP profiles throughout the production cycle [6,27,28,29].

### 1.3. Aim of the Review

The purpose of this review is to summarise, in a comparative manner, recent advances in LEP biology, with particular emphasis on its role in the regulation of reproduction in dairy cattle. The review cites studies on species other than cattle, both to highlight the cellular mechanisms of LEP and to emphasise the differences between these species and the cow.

## 2. Metabolic Pathways of Leptin

### 2.1. Leptin Synthesis, Receptor Isoforms and Signalling Pathways

The source of LEP and its target tissues are well characterised in cattle: LEP is synthesised as a preprotein of 167 amino acids (AA) almost exclusively in adipose tissue and circulates as a mature protein of 146 AA after removal of the signal peptide [14].

The LEP receptor (ObR) has a single membrane-spanning domain and exists in different isoforms (ObRa, ObRb, ObRc, ObRd, ObRe, and ObRf) that derive from alternative splicing of mRNA [30]. All isoforms have similar ligand-binding domains, but differ at the C-terminus of the intracellular domain [31]. ObRb, which contains a long intracellular domain, is the only isoform with both protein motifs necessary for activation of the Janus kinase 2 (JAK2) and signal transducers and activators of transcription 3 (STAT3) pathway [32,33]. Although the JAK2/STAT3 pathway has been considered the main signalling mechanism activated by ObR, mitogen-activated protein kinase (MAPK) [34] and phosphatidylinositol-3 kinase (PI3K) [33,34] have also been implicated in ObR signalling.

The hypothalamus is an important structure in the regulation of energy intake [17]. Alpha-1 acid glycoprotein (AGP), an acute phase protein that suppresses food intake in rodents by binding to hypothalamic ObR and activating the JAK2/STAT3 pathway [18,35], does not exert the same effects in cattle [15,16]. These latter studies demonstrate the absence of AGP effects on STAT3 signalling and food intake in cattle, revealing important species-specific differences in LEP signalling mechanisms. This divergence probably reflects evolutionary adaptations in bovine energy homeostasis, where the multicompartment digestive system and reliance on ruminal fermentation require distinct neuroendocrine regulatory pathways.

### 2.2. Tissue Distribution of Leptin Receptors in Cattle

The distribution of ObR isoforms in dairy cattle has been surveyed by measuring all LEP receptor isoforms (ObRTOTAL), ObRb, and ObRa [36]. The liver had the highest expression of ObRTOTAL among the 20 tissues surveyed; significant expression was also found in all other tissues, including adipose tissue, muscle, and mammary parenchyma. Non-signal ObRa represented almost all ObRTOTAL in these and other peripheral tissues [37].

## 3. Leptin Production and Regulation

### 3.1. Sources of Leptin in Dairy Cattle

In dairy cattle, adipose tissue is the primary source of circulating LEP, and the expression of the LEP gene in fat depots is a major determinant of plasma LEP concentrations [28]. The positive effect of increasing the nutritional level on serum LEP has been documented in Holstein heifers [38,39], where higher dietary energy and protein supply promoted greater body fat deposition and increased LEP concentrations. Body weight and back fat thickness together explain a substantial proportion of the variability in LEP levels in growing heifers, strengthening the tight coupling between adiposity and LEP secretion in bovine females [40,41].

In humans, the LEP gene promoter appears to include a placenta-specific enhancer, and placental LEP production contributes markedly to maternal LEP during gestation [42]; on the contrary, studies in ruminants indicate negligible placental LEP expression and minimal LEP binding activity in the plasma of pregnant cows [43]. In dairy cattle, loss of placental tissue does not significantly affect circulating LEP, and its concentration begins to decrease approximately 30 days before calving [44], despite continued pregnancy. Therefore, the available evidence suggests that the gestational increase in plasma LEP in ruminants is largely explained by the increased expression of LEP mRNA in adipose tissue and progressive adiposity, while placental LEP appears to play only a minor role in shaping maternal LEP profiles in the late gestation [45].

### 3.2. Nutritional and Hormonal Regulation of Leptin

Nutritional management and body condition score (BCS) are the main determinants of LEP secretion in dairy cows and heifers [46]. During the transition period, several studies have reported that LEP concentrations are highest in late gestation, drop dramatically during the peripartum period, and remain low during early lactation, when cows typically experience negative energy balance (NEB) and elevated non-esterified fatty acids (NEFA) [47,48]. Studies [39,49] on heifers and Holstein calves have shown that higher pre- and postweaning nutritional protocols increase LEP concentrations, consistent with increased adipose accretion. In these animals, improved energy and protein supply before and after weaning led to greater body fat deposition and greater circulating LEP [38,40], which were associated with more favourable endocrine profiles for growth and reproductive development [41].

Hormonal factors mediate part of the variation in LEP expression. In vivo studies indicate that the administration of glucocorticoid and insulin can increase the expression of the LEP gene [50,51], suggesting that both hormones act as upstream regulators that link dietary energy intake with the LEP output of adipose tissue. In transition dairy cows, hyperinsulinemic—euglycemic clamp experiments demonstrated that insulin can increase circulating LEP at multiple stages of the pregnancy—lactation cycle [51,52]. These studies support the causal role of insulin in maintaining higher levels of LEP in energy-replete states. In contrast, the development of hypoinsulinemia at the onset of lactation is closely followed by a decrease in plasma LEP [53], and this reduction is further amplified by up-regulation of the nonsignaling ObRa isoform in peripheral tissues, which likely enhances receptor-mediated LEP clearance [54,55].

Sex also influences LEP profiles, with circulating LEP concentrations generally lower in men than in females [56,57], according to differences in adiposity and the endocrine environment. In heifers and adult cows, serum LEP tends to decrease during the late luteal and early follicular phases of the estrous cycle, reflecting reduced expression of the LEP gene in adipocytes and suggesting dynamic short-term modulation of LEP secretion throughout the ovarian cycle [58].

### 3.3. Leptin and Milk Production

The transition from late gestation to early lactation in high-yielding dairy cows is characterised by a rapid increase in milk production, a marked increase in energy requirements, and the development of NEB, often in the absence of substantial initial loss of body condition [27,29,59]. Under these conditions, plasma LEP concentrations decrease in parallel with the onset of hypoinsulinemia, suggesting that reduced insulin drive to adipose tissue is a primary cause of the early postpartum decrease in LEP [27]. Hyperinsulinemic–euglycemic clamp studies confirm that insulin stimulates LEP secretion in dairy cows [54,60], and the persistence of elevated LEP after calving when milk synthesis is experimentally prevented further supports the central role of insulin, rather than milk production per se, in maintaining higher LEP levels [27,54,55].

At the same time, early lactation is accompanied by increased GH concentrations and a progressive uncoupling of the GH-IGF-1 axis, GH that promotes lipolysis and antagonises insulin action in adipose tissue [27,61,62]. This endocrine configuration, together with up-regulated expression and increased receptor-mediated LEP clearance, contributes to lower circulating LEP and facilitates lipid mobilisation to support milk production [27]. From an evolutionary and functional perspective, the fall in LEP during early lactation appears to be part of a coordinated adaptation that prioritises the allocation of nutrients to the mammary gland while reducing energy expenditure in non-mammary tissues [63,64].

Contrary to the situation in monogastric species, where a drop in LEP typically signals nutritional deficiency and increases appetite, available data indicate that changes in physiological plasma LEP concentrations have limited impact on short-term feed intake in ruminants [20]. Intravenous infusion of human LEP to restore early lactation cows to late gestation LEP levels did not reduce dry matter intake (DMI) or milk energy production [19], and peripheral administration of LEP has failed repeatedly to generate anorexigenic responses in sheep and cattle [21,23]. Appetite suppression has been observed only after intracerebroventricular administration of supraphysiological LEP doses [21,23], and even this central effect disappears under chronic energy deficiency, such as that experienced in early lactation [22,24]. Together, these findings support the view that, in dairy cows, LEP acts primarily as a long-term metabolic signal rather than as an acute satiety factor, contributing to the regulation of glucose and lipid partitioning, thyroid function, and immune activity during the transition period [15,16,18,65,66].

## 4. Leptin and the Hypothalamic–Pituitary–Gonadal Axis

### 4.1. Central Leptin Signalling and GnRH/Gonadotropins Regulation

Studies on ObRb expression in cattle indicate that the brain is the primary site of LEP action, with the hypothalamus showing the highest transcription among the tissues examined, which is consistent with observations in other mammals [36]. Within the bovine hypothalamus, ObRs are located in the nuclei involved in the regulation of food intake, energy expenditure and reproductive function, placing LEP in a strategic position to connect metabolic status to activity of the hypothalamic-pituitary-gonadal (HPG) axis [36].

Sexual maturation and nutritional status are the main determinants of LEP effects on the hypothalamic–hypophyseal axis in ruminants (Table 1) [67,68,69]. Comparative studies in mammalian species, including cattle, indicate that the long ObRb isoform is expressed in ventromedial and arcuate hypothalamic nuclei and in the anterior pituitary, where LEP influences the neuronal networks that control gonadotropin secretion [67,68,69]. These networks involve several neuropeptides, such as products derived from neuropeptide Y (NPY), proopiomelanocortin (POMC) derived products, γ-aminobutyric acid (GABA) and cocaine and amphetamine-regulated transcript (CART), which integrate signals of energy balance and modulate the release of gonadotropin-releasing hormone (GnRH)/LH [70,71,72,73,74,75,76,77]. Although direct actions of LEP on GnRH neurones have been proposed, current evidence favours an indirect mechanism mediated largely by these intermediary neuronal populations, with NPY and POMC/α-melanocyte-stimulating hormone (α-MSH) playing prominent roles [70,78,79,80,81,82].

In farm animals, periods of metabolic stress are associated with increased hypothalamic NPY expression, reduced pulse frequency, and stimulation of feed intake, supporting a model in which activation of NPY pathways contributes to the processes of reproductive suppression observed during undernutrition [71,72,83]. Leptin, by signalling adequate energy reserves, is believed to counterbalance this inhibition and favour the resumption or maintenance of pulsatile GnRH and LH secretion, particularly in nutritionally challenged animals [114].

Functional studies in ruminants have shown that LEP can modulate gonadotropin secretion, especially under conditions of nutritional stress [115]. In prepubertal heifers subjected to short-term fasting, LEP treatment prevented the fasting-induced reduction in LH pulse frequency, indicating that LEP can maintain hypothalamic drive to the pituitary when energy availability is compromised [115]. In contrast, in mature cows adequately fed, short-term fasting is often insufficient to suppress the pulse frequency of LH, and LEP administration in this context primarily increases the basal and mean LH concentrations by increasing the pulse amplitude rather than the frequency [116].

In early lactation dairy cows, delayed or failed ovulation and reduced fertility are commonly observed and have been partially attributed to suppression of pulsatile LH secretion in the presence of NEB [84]. Given the inverse association between plasma LEP and NEB, hypoleptinemia has been proposed as a candidate mediator of these reproductive defects [19]. Observational studies in early lactation cows have reported positive relationships between plasma LEP levels, LH pulsatility, and a shorter interval to the first postpartum oestrus, supporting this hypothesis [44]. Furthermore, continuous infusion of LEP at physiological concentrations in energy-deficient or early-lactation cows was able to maintain significant pulsatility of LH, suggesting that LEP contributes to maintaining GnRH/LH pulse generation under challenging metabolic conditions [19,22,116].

In general, the stimulatory effect of LEP on LH secretion in ruminants appears to be confined mainly to periods of nutritional stress, when endogenous LEP concentrations are low and the HPG axis is particularly sensitive to metabolic signals [115,116]. Direct actions of LEP at the adenohypophyseal level have also been demonstrated in cattle, although the molecular pathways responsible for increased pituitary sensitivity to LEP in undernourished animals have not yet been fully elucidated [117]. In particular, LEP-induced hypersecretion of LH in fasted cows is not associated with increased ObR mRNA or reduced suppression of cytokine signalling 3 (SOCS3) expression in the anterior pituitary, indicating that post-receptor mechanisms likely explain these changes [85].

### 4.2. Relationship with Ovarian Steroids

Plasma LEP concentrations increase as dairy heifers approach puberty and show higher circulating oestrogen levels, consistent with the permissive role of LEP in supporting reproductive maturation [56,57]. However, studies [36,96] on ovariectomised prepubertal heifers indicate that 17β-estradiol does not significantly affect ObR expression of ObR in the hypothalamus, liver, skeletal muscle, or subcutaneous adipose tissue. These studies indicate that the increase in oestrogen during pubertal development does not directly drive changes in the abundance of ObRs in these tissues. Instead, estradiol may modulate ObR expression in selected estrogen-sensitive targets, potentially fine-tuning tissue response to LEP without altering systemic LEP concentrations [36,96].

In adult cows, circulating LEP shows modest fluctuations throughout the estrous cycle, with lower levels generally observed during the late luteal and early follicular phases, in parallel with reductions in LEP gene expression in adipose tissue [58]. These short-term changes occur within the larger context of long-term regulation by body condition and energy balance, indicating that LEP integrates both chronic (adiposity-related) and cyclic (ovarian-related) influences in shaping its impact on the reproductive axis of dairy cattle [56,57,58].

## 5. Leptin in Ovarian and Testicular Function

### 5.1. Ovarian Leptin Receptors and Steroidogenesis

Leptin participates in the control of reproductive function not only through its central actions on the hypothalamic–pituitary axis but also through its direct effects on the gonads [86]. Leptin receptors have been identified in bovine ovaries, indicating that locally produced LEP can modulate ovarian function at multiple levels [86]. Bovine granulosa and theca cells express high-affinity ObRs and in vitro studies have shown that LEP can influence their steroidogenic activity, thus affecting estradiol and progesterone production [118]. Furthermore, LEP signalling in the ovary may influence oocyte competence, improving oocytes’ ability to maintain subsequent embryonic development under certain experimental conditions [86,118].

### 5.2. Leptin in Folliculogenesis and Ovulation

The role of LEP in the ovarian follicular dynamics is complex and, in some respects, controversial. In livestock, several studies have associated high concentrations of circulating LEP, or sustained hyperleptinemia, with inhibitory effects on ovarian steroidogenesis, consistent with in vitro evidence that LEP can suppress estradiol and progesterone synthesis in bovine granulosa and theca cells at relatively high doses [118]. In sheep, chronic elevation of LEP has been reported to increase GH secretion, antagonise IGF-1 and insulin, and reduce estradiol production, ultimately altering dominant follicle development and oocyte maturation [88]. On the contrary, low levels of postpartum LEP, caused by severe NEB, compromise gonadotropin secretion and prolong ovulation time to ovulation in dairy cows [13,44,87,119], suggesting that LEP deficiency can also be detrimental to optimal follicular function. Intervention studies further highlight this duality: recombinant LEP during postpartum anestrus in beef cows with marked NEB and hypoleptinemia increased circulating oestrogen and follicle stimulating hormone (FSH) concentrations, increased follicular growth, and advanced onset of oestrus, indicating beneficial effects under conditions of LEP deficiency [13,44,87,119]. However, more recent data in cattle suggest that LEP may not be strictly essential for steroidogenesis or follicular growth, pointing instead to a modulatory role that depends on metabolic context, LEP dose, receptor expression, and interactions with other endocrine factors [119]. Because plasma LEP concentrations closely reflect the status of NEB, some authors have proposed that adipokines, including LEP, participate in the regulation of the pulse frequency of LH and the timing and amplitude of the pre-ovulatory surge of LH in dairy cows [13,118,120]. In sheep, for example, increases in plasma LEP have been observed in parallel with a higher pulse frequency, supporting a functional link between LEP, gonadotropin secretion, and follicular maturation [88,121].

### 5.3. Leptin, Oocyte Competence and Early Embryonic Development

Leptin is also involved in the regulation of oocyte maturation and early embryo development [86]. Leptin receptors have been detected in bovine embryos [86], and the addition of LEP to the in vitro maturation medium has been reported to improve meiotic progression, increase blastocyst yield, and improve embryo quality in calf oocytes [86,109,110]. Furthermore, LEP administration during superovulation protocols has been associated with improved embryo quality in some domestic species, suggesting that carefully controlled exposure to LEP around the periovulatory period may support oocyte competence and early embryogenesis [108].

### 5.4. Leptin in Testicular Function

Although less extensively studied than in females, LEP and its receptors are also expressed in testicular tissue, where they are believed to participate in the regulation of steroidogenesis and spermatogenesis [122]. Both Leptin and ObR are present in the bovine testes, suggesting that LEP is involved in autocrine and/or paracrine mechanisms in testicular physiology in cattle [123]. Taken together, the female and male observations support the concept that LEP acts as a peripheral metabolic signal to ovarian and testicular cells, integrating systemic energy balance with local gonadal activity in cattle.

## 6. Leptin and Uterine Function

### 6.1. Impact on Uterine Receptivity and Embryo Development

Beyond its ovarian actions, LEP appears to influence uterine function and embryo–maternal interactions. Successful embryonic development depends not only on nuclear maturation, but also on cytoplasmic maturation of the oocyte, which involves extensive organelle reorganisation and accumulation of mRNA, proteins, and transcription factors [92]. In cattle, in vitro-matured calf oocytes often exhibit suboptimal cytoplasmic maturation compared to adult oocytes, resulting in reduced developmental competence [92]. Supplementation of in vitro maturation media [92] with LEP has been shown to improve cytoplasmic maturation and increase the proportion of embryos that develop to the blastocyst stage, indicating a supporting role for LEP in early embryogenesis [110].

### 6.2. Immune Modulation in the Reproductive Tract

Leptin also acts as an immunomodulatory cytokine within the reproductive tract and has been implicated in uterine inflammatory disorders in dairy cows [112,124,125]. In general, LEP exerts predominantly pro-inflammatory actions on immune cells, but in states of severe energy deprivation, very low LEP can also contribute to immunodeficiency. In rodent models, replacement with LEP partially restores immune function under starvation, illustrating the context-dependent nature of its immunomodulatory effects [107,126,127]. Recent clinical studies report higher serum LEP concentrations in cows with postpartum pyometra compared to healthy controls, as well as a decrease in LEP after successful treatment, and document moderate to high diagnostic precision of LEP as a biomarker of this condition in the specific study populations examined [112,124,128]. Similarly, cows with clinical metritis or endometritis exhibit significantly elevated LEP levels compared to healthy animals, and LEP concentrations correlate positively with the severity of uterine inflammation, suggesting that systemic inflammation and local uterine disease enhance LEP production by adipose tissue and possibly by reproductive tissues themselves [124,125,128]. Because LEP is closely related to both metabolic status and inflammatory tone, its elevation in uterine disease probably reflects the combined influence of systemic energy balance, adipose-derived cytokine release, and local immune activation [91,128]. Taken together, the current evidence supports the use of LEP, alone or in combination with other adipokines and inflammatory markers, as a promising component of multimarker panels for postpartum uterine disorders and as a potential target for strategies aimed at improving uterine health and fertility in dairy herds, while recognising that LEP is unlikely to serve as a standalone diagnostic test [89,90,91,112,124,125].

## 7. Metabolic Status, Peripartum Period, and Fertility

### 7.1. Relationship Between Leptin, Energy Balance, and Fertility

In high-yielding dairy cows, the transition from late gestation to early lactation is characterised by NEB, intense lipid mobilisation, and major endocrine adaptations that are closely related to fertility [52,129]. Recent studies [91,130] examining postpartum ovarian inactivity reported lower circulating concentrations of LEP and other cytokines in transition cows with inactive ovaries compared to cyclic animals, consistent with the role of adipokines in modulating insulin sensitivity, metabolic status, and reproductive function. In these cows, composite indices, including LEP and related cytokines, have shown high sensitivity and specificity to predict reproductive alterations, supporting their potential use as early warning biomarkers [91,130] to guide timely interventions and improve reproductive outcomes at the herd level [52,130].

Although no direct association is consistently demonstrated between LEP and the timing of the first postpartum luteal activity, higher LEP concentrations have been associated with shorter intervals to the first observed oestrus and faster resumption of ovarian cyclicity in dairy cows [44]. In contrast, reduced postpartum LEP has been associated with delayed ovarian rebound and prolonged open days, highlighting the importance of adequate energy status and LEP signalling for optimal reproductive performance. Therefore, integration of LEP measurements with other metabolic indicators during the transition period can improve early detection of cows at risk of subfertility and support more targeted reproductive management strategies [91,130].

### 7.2. Leptin and the Peripartum Period

Leptin, together with adiponectin, is highly concentrated in bovine colostrum, and both hormones are absorbed from the intestine of neonatal calves during the first hours of life. Colostrum-fed calves show marked postcolostrum increases in plasma LEP and adiponectin, while formula-fed calves exhibit flat or blunt profiles, demonstrating that the early postnatal surge in these adipokines originates from colostral uptake rather than endogenous synthesis [49,111]. These hormones remain elevated for several days after birth and are known to improve insulin sensitivity, modulate glucose homeostasis, and support intestinal development, suggesting that colostral LEP and adiponectin contribute to metabolic programming in neonatal calves [131,132].

Studies comparing colostrum feeding versus formula feeding have shown that, despite similar plasma glucose concentrations, formula-fed calves have higher early postnatal insulin levels, likely reflecting higher lactose intake and the absence of colostral LEP and adiponectin [49,111]. Low LEP and adiponectin in formula-fed calves can alter insulin regulation, contributing to transient hyperinsulinemia, while in colostrum-fed calves, LEP can dampen pancreatic insulin release and improve insulin efficiency [122,131,133,134,135]. These findings highlight the potential long-term implications of early postnatal exposure to adipokine for metabolic health and growth performance in dairy replacements [131,133,135].

### 7.3. Leptin and Peripartum Ketosis

Leptin is a key component of the adipose–hypothalamic–hepatic axis that regulates appetite, fat mobilisation, and insulin sensitivity during the peripartum period [136,137]. Serum LEP concentrations are directly proportional to body fat stores and are inversely related to DMI, and LEP can inhibit lipogenesis, promote triglyceride hydrolysis, reduce fatty acid synthase, and decrease insulin sensitivity of adipose tissue [136]. In periparturient cows, LEP concentrations in both healthy animals and cows that later develop ketosis are relatively stable before calving, but decrease after parturition and gradually recover in about four weeks, reflecting intense fat mobilisation and NEB adaptation [136,138]. Cows with clinical ketosis exhibit lower postpartum LEP concentrations than healthy controls, likely as a consequence of increased fat mobilisation and altered insulin and energy status [138].

Although LEP is not a direct causal factor in ketosis, its dynamics mirror changes in lipid mobilisation and can modulate the risk of excessive accumulation of non-esterified fatty acids and ketone bodies through its actions on appetite, adipose metabolism, and liver function [136,138]. Lower LEP concentrations in ketotic cows may represent both a marker of severe NEB and a permissive signal that facilitates further lipid mobilisation, thus contributing to a self-reinforcing metabolic imbalance [138]. In this context, the monitoring of LEP, together with classical metabolic indicators such as NEFA, β-hydroxybutyrate, and insulin, could improve early identification for cows at high risk of peripartum metabolic disease [136,138].

## 8. Leptin in the Metabolic Adaptation of Early Lactation

As detailed in Section 3.3, the transition to lactation is characterised by a marked decline in plasma LEP concentrations associated with hypoinsulinemia and NEB rather than an overt loss of body condition. [27,29,54,55,59,60]. Insulin is an important upstream regulator of LEP secretion in this phase, while increased GH concentrations, uncoupling of the GH-IGF-1 axis, and enhanced receptor-mediated LEP further contribute to a lower circulating LEP and to lipid mobilisation to support of milk production [8,9,27,54,55,61,62]. In monogastric species, decreased LEP is a powerful signal of energy deficiency that increases appetite and triggers central metabolic adaptations, but in ruminants physiological changes in LEP appear to have limited impact on short-term feeding intake. Intravenous treatment of LEP during late gestation did not reduce energy intake or milk production, and peripheral administration of LEP or the ObR antagonist altered feed intake in sheep and cows under various nutritional conditions [22,23,24,25]. In dairy cows, these changes appear to have limited effects on short-term food intake, supporting the view that LEP acts mainly as a long-term metabolic signal that coordinates nutrient partitioning, thyroid function, and immune activity during early lactation [15,16,18,19,20,23,27,61,62,65,66]. This interpretation is also consistent with the broader discussion in Section 7 on the functional importance of LEP in the transition period and its relationship with metabolic and reproductive outcomes.

## 9. Leptin, Growth, and Puberty

Leptin plays a crucial role in the association of nutritional status, body growth, and maturation of the neuroendocrine reproductive axis in cattle [58,139]. In growing heifers, circulating LEP concentrations are positively associated with adiposity and gain in body weight of LEP, and increases are generally observed as animals approach puberty [58]. Experimental studies in *Bos indicus* and *Bos taurus* heifers have shown that higher nutritional planes, leading to greater energy and protein intake, increase LEP secretion and are associated with earlier onset of puberty [140]. These findings support the concept of a ‘LEP threshold’ that signals sufficient somatic growth and energy reserves to initiate reproductive activity [139].

Adult bull biostimulation has been shown to advance puberty in Bos indicus heifers, and this effect has been accompanied by longer feeding times, greater weight gain, and higher circulating concentrations of LEP and GH in biostimulated animals [141]. Although the mechanisms by which bull exposure enhances LEP and GH secretion remain incompletely understood, the known stimulatory effects of both hormones on growth and reproductive maturation provide a plausible link between biostimulation and earlier puberty [139,140,142]. Interestingly, some studies have reported increased LEP secretion in biostimulated heifers without marked differences in body weight or body condition, suggesting that LEP may also mediate more direct effects of social or pheromonal signals on the reproductive axis, in addition to reflecting changes in adiposity [143,144,145,146,147]. Comparable observations in mice, where exposure of females to male pheromones increased GH secretion and accelerated growth and reproductive maturation, further support a conserved connection between environmental signals, somatotropic hormones, and puberty timing in mammalian species [141,144,147,148]. However, in cattle the relative contribution of direct effects of biostimulation to GnRH/LH secretion versus indirect effects mediated by metabolic hormones such as LEP and GH has not yet been fully clarified and remains largely inferential [142,143,145,146]. The nutritional plan during the pre- and post-weaning periods also exerts significant effects on LH pulsatility and pubertal development [39,40,149]. In Holstein heifers, increased preweaning nutrition has been associated with greater amplitude of LH pulses, higher LH peaks, and longer LH pulses around 15 weeks of age, although the effects on LH pulse frequency were less consistent [39]. Beef heifers fed higher energy postweaning diets showed an increased LH pulse frequency of LH during intensive sampling immediately before puberty, but not at earlier ages, indicating that nutritional effects on LH secretion become more evident as heifers approach sexual maturity [149].

In all studies, higher-fat or higher-energy diets have generally reduced age of puberty onset, consistent with a coordinated increase in LEP, improved growth, and earlier activation of the GnRH–LH axis in growing heifers [39,139,140,149]. Collectively, these data support a model in which LEP acts as a key metabolic signal that integrates nutritional input, body composition, and environmental stimuli, such as biostimulation, to modulate GH secretion, LH pulsatility, and the timing of puberty in dairy and beef heifers [41,58,95,97,139,140].

## 10. Birth Weight, Placental Leptin, and Intrauterine Growth

Compared to humans and rodents, data on placental adipokines and intrauterine growth in cattle remain limited, but emerging evidence suggests that LEP may contribute to fetoplacental development and birth weight [94,105]. In cattle, placental LEP expression appears lower than in primates and rodents, and most mechanistic insights into trophoblast proliferation, migration, and JAK–STAT or MAPK signalling derive from non-ruminant models and should therefore be extrapolated with caution [93,99,104]. In dairy calves, placental LEP expression has been positively correlated with total birth weight, according to studies in women and experimental animals linking higher placental LEP with increased foetal growth [104,105]. Bovine trophoblast cells express LEP and its receptors, and work in other species indicates that LEP can promote trophoblast proliferation and migration, modulate amino acid transport, inhibit apoptosis, and support placental formation [93,99]. Placental and foetal LEP signalling appears to regulate foetal growth and skeletal development and to reduce developmental arrest in experimental models, but the relative contribution of these pathways in cattle is still poorly defined [96,100,102]. High concentrations of placental LEP have been associated with improved foetal growth and a lower risk of reduced intrauterine development in several species, including cow [94,98,101,103,106,150,151,152]. In cattle, greater expression of placental LEP in heavier calves may represent a local compensatory mechanism to enhance prenatal growth when foetal size tends to be low, although exact mechanisms and potential trade-offs for dystocia or postpartum health require further investigation [104,105].

## 11. Genetic Selection Based on Leptin Polymorphisms and Feed Efficiency

Genetic variation in the bovine LEP gene has attracted considerable interest due to its associations with milk production, energy balance, feed intake, and fertility traits [153,154]. The LEP gene is located on chromosome 4, and early sequencing efforts identified multiple polymorphisms across exons and introns in diverse cattle populations [153,155]. Among these, the g.-963C>T mutation in the LEP promoter and the c.357C>T single nucleotide polymorphism (SNP) in exon 3 (alanine to valine substitution) have been studied more extensively in relation to reproductive performance and milk production [155,156,157,158]. The g.-963C>T TT genotype has been associated with earlier age at first calving and trends towards shorter open days and higher pregnancy rate, while the c.357C>T TT genotype has been associated with longer calving intervals and reproductive periods, suggesting that g.-963C>T TT may be favourable and c.357C>T TT unfavourable to fertility [120,156,157,158,159].

LEP polymorphisms have also been associated with food intake, milk production, somatic cell count, and milk composition, indicating pleiotropic effects on production and health traits [155,157,158]. Genome-wide and candidate gene studies of residual feed intake (RFI) and DMI in dairy cows have identified quantitative trait loci (QTL) on chromosome 4 that include LEP, as well as other chromosomes that harbour genes such as β-3 adrenergic receptor (ADRB3), supporting the role of LEP and its signalling network in the genetic regulation of feed efficiency [160]. Transcriptomic comparisons between low and high RFI cows showed a decrease in the regulation of LEP and components of the LEP–NPY signalling pathway in more efficient animals, suggesting that reduced leptinergic drive may contribute to lower voluntary intake and improved efficiency without compromising milk production [161,162,163]. These findings illustrate the physiological complexity of feed efficiency and support the incorporation of LEP genotypes and related markers into genomic selection programmes aimed at simultaneously improving productivity, fertility, and metabolic robustness in dairy cattle [44,160,161,162,163].

## 12. Leptin as a Biomarker in Dairy Practice

Leptin has been increasingly investigated as a candidate biomarker at the interface between metabolism and reproduction in dairy cows (Table 2) [89,90,91,125]. Circulating LEP concentrations reflect body fat reserves and changes in energy balance, particularly during the transition period, and have been associated with subclinical ketosis, insulin resistance, and altered insulin sensitivity [91]. Cows that develop periparturial metabolic disorders often show lower or more pronounced declines in LEP, in parallel with elevated levels of NEFA and ketone bodies, suggesting that the dynamics of LEP can help identify animals at risk before clinical disease becomes evident [89,90,124,125].

In the reproductive domain, reduced postpartum LEP levels have been associated with ovarian inactivity, delayed resumption of cyclicity, and longer intervals to the first oestrus, while higher LEP concentrations are associated with more favourable reproductive performance [44,91,130]. Elevated LEP has also been reported in cows with uterine inflammation, including metritis, endometritis, and pyometra, where LEP levels correlate with the severity of uterine lesions and show good diagnostic sensitivity and specificity [52,124,125,129]. These findings indicate that low and high LEP values, interpreted in the appropriate physiological context, can provide clinically relevant information on reproductive health [44,91,130].

Given the multifactorial nature of transition cow disorders, LEP alone is unlikely to serve as a standalone diagnostic test, but its inclusion in multimarker panels alongside NEFA, β-hydroxybutyrate (BHB), insulin, inflammatory markers, and other adipokines may improve the prediction of metabolic and reproductive problems [89,90,91,124,125]. From a practical point of view, strategic LEP measurements in late gestation and early lactation, combined with body condition scoring and routine metabolic profiling, could support precision management of high-risk cows and inform decisions on nutrition, reproduction and health interventions at both the individual and herd level [52,91,129,130].

## 13. Conclusions and Future Perspectives

Leptin is a key biomarker at the interface between metabolism and reproduction in dairy cows, especially during the transition period [27,29,59]. Circulating LEP reflects body fat reserves and energy balance and has been linked to subclinical ketosis, insulin resistance, postpartum ovarian inactivity, and uterine inflammatory disease [27,29,44,59,91,112,124,125,128]. LEP improves risk stratification when interpreted together with classical metabolic and inflammatory indicators (NEFA, β-hydroxybutyrate, insulin, acute phase proteins, other adipokines), while its value as a standalone diagnostic test appears limited [89,90,91,112,124,125]. Polymorphisms in the LEP gene and its signalling pathway have been associated with milk production, body condition, feed efficiency, and fertility traits, indicating that these markers could be incorporated into genomic selection programmes to improve robustness and reproductive performance without compromising productivity [52,129,137]. Integrating LEP genotypes with phenotypic indicators of metabolic status and fertility can help identify animals that better tolerate peripartum metabolic challenges while maintaining reproductive function under intensive production conditions [52,129,130]. Future research should validate LEP and other adipokines as elements of multimarker panels for the early prediction of metabolic and reproductive disorders in breeds, housing systems, and nutritional strategies [52,91,129,130]. Studies are also needed to clarify species-specific aspects of LEP signalling in ruminants, including its limited acute anorexigenic effects and its direct versus indirect roles in puberty, folliculogenesis, uterine immunity, and peripartum inflammatory diseases [19,21,22,23,24,25,44,56,57,58,86,88,112,118,124,125,128,129].

Ultimately, a more integrated understanding of the biology of LEP in dairy cows will be able to support the design of combined nutritional, reproductive and genetic interventions that improve animal health, welfare and farm profitability in modern dairy production systems [15,16,19,27,29,61,62,129,130].

## Figures and Tables

**Table 1:** Summary of the physiological roles, signalling pathways, and clinical significance of leptin in diverse organ systems, involved in metabolic and reproductive health.

Target Tissue	Specific Effects of Leptin	Biological Pathway	Clinical Significance	References
Hypothalamus	Regulates the release of GnRH Transmits satiety signals via NPY, POMC, and GABA mediation Acts on ventromedial and arcuate nuclei	JAK2-STAT3 MAPK PI3K	Critical link between energy balance and reproductive health Regulates puberty onset and LH pulsatility	[67,69,70,71,72,73,74,75,76,77,78,79,80,81,82,83]
Anterior Pituitary	Enhances basal and GnRH-mediated release of LH Regulates gonadotropin secretion	JAK-STAT MAPK	Impacts fertility during nutritional stress Prevents reduction in LH pulse frequency during fasting	[56,57,58,71,72,73,74,84]
Ovarian follicles: Granulosa and Theca cells	Modulates steroidogenesis (estrogen and progesterone production) Influences folliculogenesis, oocyte maturation, and ovulation	IGF-1	Low levels associated with postpartum anestrus and reproductive failure High levels may inhibit steroidogenesis	[17,35,85,86,87,88]
Uterus	Modulates immune responses and uterine receptivity Impacts embryo development and implantation	Inflammatory cytokine signalling	Correlation between leptin levels and disease severity	[89,90,91,92]
Placenta	Promotes trophoblast cell migration and proliferation Inhibits apoptosis Facilitates placenta formation	JAK-STAT	Positive correlation with high-birth-weight neonates Prevents intrauterine growth retardation Essential for fetomaternal communication	[93,94,95,96,97,98,99,100,101,102,103,104,105,106]
Mammary Gland	Upregulates the lactogenic effect of prolactin Expression found in mammary parenchyma	Lactogenic signalling upregulation	Potential impact on milk production and composition during early lactation	[18,60,107]
Adipose Tissue	Inhibits fat synthesis Promotes triglyceride breakdown Reduces insulin sensitivity in adipocytes	Autocrine/Paracrine signalling	Regulation of lipid metabolism	[16,21,22,24,51,88,91]
Liver	Modulates insulin-mediated glucose clearance Exhibits the highest expression of total leptin receptors	JAK2-STAT3	Involved in glucose conservation during the transition from pregnancy to lactation	[4,6,7,13,16,46,47,49,95,107,108,109,110,111,112,113]
Skeletal Muscle	Increases glucose uptake Determinant of energy expenditure and thyroid hormone-mediated thermogenesis	Autonomic nervous system activation	Effector tissue for energy sparing in transitioning dairy cows Impacts metabolic adaptation during weight loss	[62,64,106]

**Table 2:** Leptin as a biomarker in dairy practice.

Domain	Leptin Signal	Key Message	Practical Implication	References
Transition energy balance	Circulating leptin reflects body fat reserves and changes in energy balance during the transition period.	Leptin serves as an indicator of metabolic adaptation around calving.	Enables early identification of metabolically fragile cows in late gestation and early lactation.	[91]
Periparturient ketosis	Lower or sharply declining leptin is associated with elevated NEFA and ketone bodies in cows that develop ketosis.	Low leptin signals, severe NEB and intense lipid mobilisation.	Inclusion of leptin in metabolic screening may improve early detection of cows at risk of ketosis.	[136,137,138]
Postpartum fertility	Reduced postpartum leptin is linked to ovarian inactivity, delayed resumption of cyclicity, and longer interval to first oestrus.	An unfavourable leptin profile indicates impaired coupling between metabolic status and reproductive function.	Leptin measurements can help flag cows at risk of subfertility during the transition period.	[44,91,130]
Uterine inflammatory disease	Higher leptin concentrations are reported in cows with metritis, endometritis, or pyometra and correlate with disease severity.	In this context, leptin behaves as an immunometabolic marker of uterine inflammation rather than merely an energy-status marker.	Supports diagnosis and monitoring of uterine disease when interpreted within multimarker panels.	[52,124,125,129]
Biomarker strategy	Leptin shows good performance but is insufficient as a standalone diagnostic test.	Its clinical value increases when combined with NEFA, BHB, insulin, inflammatory markers, and other adipokines.	Recommended use is as part of integrated biomarker panels for precision management at cow and herd level.	[89,90,91,124,125]

## Data Availability

No new data were created or analyzed in this study. Data sharing is not applicable to this article.

## Abbreviations

The following abbreviations are used in this manuscript:

AA	Amino acids
α-MSH	α-Melanocyte-stimulating hormone
ADRB3	β-3 adrenergic receptor
AGP	Alpha-1-acid glycoprotein
BCS	Body condition score
BHB	β-Hydroxybutyrate
CART	Cocaine and amphetamine-regulated transcript
DMI	Dry matter intake
FSH	Follicle-stimulating hormone
GABA	Gamma-aminobutyric acid
GH	Growth hormone
GnRH	Gonadotropin releasing hormone
HPG axis	Hypothalamic–pituitary–gonadal axis
IGF-1	Insulin-like growth factor-1
JAK	Janus kinase
LEP	Leptin
LH	Luteinizing hormone
MAPK	Mitogen-activated protein kinase
NEB	Negative energy balance
NEFA	Non-esterified fatty acids
NPY	Neuropeptide Y
ObR	Leptin receptor
PI3K	Phosphatidylinositol-3-kinase
POMC	Proopiomelanocortin
QTL	Quantitative trait locus
RFI	Residual feed intake
SNP	Single nucleotide polymorphism
SOCS3	Suppressor of cytokine signalling 3
STAT3	Signal transducer and activator of transcription 3
